# Cell Type-Specific Proteomic Cargo in Human Brain Endothelial, Astrocyte, and Neuronal Extracellular Vesicles

**DOI:** 10.3390/proteomes14020024

**Published:** 2026-05-01

**Authors:** Hope K. Hutson, Guoting Qin, Chengzhi Cai, Gergana G. Nestorova

**Affiliations:** 1 Molecular Sciences and Nanotechnology, Louisiana Tech University, Ruston, LA 71270, USA; khh012@latech.edu; 2 College of Optometry, University of Houston, Houston, TX 77004, USA; gqin@central.uh.edu; 3 Department of Chemistry, University of Houston, Houston, TX 77004, USA; ccai@central.uh.edu; 4 School of Biological Sciences, Louisiana Tech University, Ruston, LA 71270, USA

**Keywords:** extracellular vesicles, mass spectrometry, endothelial cells, astrocytes, neurons

## Abstract

**Background:** Extracellular vesicles (EVs) mediate intercellular communication in the central nervous system and are a major source of biomarkers. This study characterizes the EV-derived proteome secreted by human endothelial brain cells (HEBCs), astrocytes, and neurons to identify cell-specific roles in intercellular communication in the brain. **Methods:** Mass spectrometry analyses of EVs and corresponding parent cells were performed to identify differentially enriched proteins. Gene Ontology (GO) analysis of statistically significant, abundantly expressed proteins between EVs and parent cells (log_2_ fold-change ≥ 2.0, *p* < 0.05) was performed to assess cell-specific functions. **Results:** Proteome analysis identified on average 932 proteins in astrocyte EVs (versus 1725 in parent cells), 1040 in HEBC EVs (versus 5451 in parent cells), and 470 in neuronal EVs (versus 578 in parent cells). The analysis indicated that astrocytes had the highest number of significantly abundant proteins (118), followed by HEBCs (24) and neurons (25). Astrocyte EVs were enriched in lipoproteins, complement factors, and protease inhibitors; HEBCs EVs in tight junction proteins, adhesion molecules, and protease regulators; and neuronal EVs in chromatin-associated histones, tubulin isoforms, and RNA-binding proteins. **Conclusions:** The proteomic signatures of EVs from different neurovascular unit cells suggest specialized roles in blood–brain barrier homeostasis, immune regulation, and synaptic and epigenetic signaling under healthy conditions. These baseline signatures provide a framework for future studies to investigate how brain cell-derived EVs may contribute to neurodegenerative disorders.

## 1. Introduction

Extracellular vesicles (EVs) are emerging as key mediators of intercellular communication and disease pathophysiology in the central nervous system (CNS). Current guidelines from the International Society for Extracellular Vesicles define EVs as lipid bilayer-enclosed particles released by cells that cannot replicate and that carry proteins and nucleic acids derived from their cells of origin [1]. EV-mediated protein transfer to recipient cells affects gene and protein expression [2,3] and serves as a critical mediator of neuronal and glial homeostasis [4]. This type of intercellular communication occurs across all brain cell types [5]. Since EVs cross the blood–brain barrier (BBB), their cargo represents a unique window into CNS physiology [6].

The mechanisms that regulate intercellular communication in the brain are poorly characterized [7]. Recent proteomic studies have shown a direct correlation between neurodegeneration and the proteomic profile of a patient’s blood [8], underscoring the need to identify protein markers of cellular origin in the brain. Current clinical diagnostics for neurodegenerative diseases such as Alzheimer’s disease and Parkinson’s disease rely on magnetic resonance imaging and positron emission tomography, which are limited in detecting molecular-level pathological changes that precede clinical symptoms [9]. This diagnostic limitation is problematic because molecular-scale changes in the brain occur years or decades before behavioral manifestations [8]. In contrast, because EVs are actively secreted from brain cells into the circulation and retain cell-of-origin molecular signatures, they provide noninvasive biomarkers for real-time monitoring of CNS status from easily accessible blood samples [10]. A recent study identified a correlation between astrocyte-derived EV-associated proteins and Alzheimer’s disease pathology and cognitive impairment, linking these findings to mechanisms underlying disease pathogenesis [11].

A fundamental requirement for identifying disease-related changes in EV composition is establishing a comprehensive baseline of cell-type-specific EV protein signatures from healthy neural tissue. EVs have selective packaging mechanisms that incorporate certain proteins while excluding others; their proteomic composition varies significantly from that of their parent cells [12]. Identification of EV-enriched proteins requires comparison between EV-derived and whole-cell proteomes. Without understanding these selective packaging mechanisms and the distinct proteomic signatures of EVs from healthy cells, it is impossible to determine whether potential biomarkers indicate an active pathological condition.

The neurovascular unit (NVU) is a multicellular complex composed of brain microvascular endothelial cells, pericytes, vascular smooth muscle cells, astrocytes, microglia, oligodendrocytes, neurons, and the surrounding extracellular matrix, which together coordinate neurovascular coupling and maintain BBB integrity [13]. The focus of this work is on three principal NVU cell types—brain endothelial cells, astrocytes, and neurons—that form the core cellular axis linking the vascular compartment to the neural parenchyma. The study characterizes their EV-derived proteomes as a baseline for cell-type-specific NVU communication. Human endothelial brain cells (HEBCs) form the structural basis of the blood–brain barrier and maintain close contact with astrocytes via astrocytic end-feet and with pericytes via the basement membrane [6]. Astrocytes provide metabolic support to neurons and maintain ionic homeostasis and synaptic function. Neurons are the functional units of the CNS and integrate signals from the broader neural network. EV communication among these cell types plays a central role in maintaining BBB integrity and function. Neurons secrete EVs that signal both to local neural circuits and, through BBB-crossing events, to the peripheral circulation [14]. The interconnected roles of these three major cell types in maintaining CNS homeostasis make them ideal candidates for baseline in vitro proteomic characterization. Despite its advantages, in vitro cultures lack three-dimensional brain architecture and in vivo signaling gradients. A well-defined in vitro baseline of cell-type-specific EV proteomes is essential for identifying cell-specific signatures. It provides a reference for future three-dimensional, multicellular in vivo complex biofluids models.

Quantitative mass spectrometry analysis of both EV and whole-cell proteomes facilitates the identification and quantification of thousands of proteins, thereby enabling selective EV enrichment [12]. This study aims to establish a proteomic baseline for EVs secreted by three major healthy brain cell types (HEBCs, astrocytes, and neurons) using quantitative mass spectrometry analysis of both EVs and their corresponding whole-cell lysates. The work identifies proteins significantly enriched in EVs relative to whole-cell lysates, thereby characterizing EV cargo subject to selective packaging. Gene ontology (GO) and pathway enrichment analyses of EV-enriched proteins were performed to connect EV cargo to biological processes. The results indicate that EVs from each cell type have distinct, selectively packaged proteomes that partially overlap with the parent cells.

## 2. Materials and Methods

### 2.1. Cell Culture

HBEC-5i Human Endothelial Brain Cells (HEBCs) (AddexBio, San Diego, CA, USA, cat. #T0005011, passage 4), Human Astrocytes (Thermo Fisher Scientific, Waltham, MA, USA, cat. Gibco™ N7805-100 passage 4), and Human Neurons (ScienCell™, Carlsbad, CA, USA, cat.#1520, passage 1) were cultured to 70–90% confluence under standardized in vitro conditions in a 5% CO_2_ incubator at 37 °C. HEBCs were seeded into a T-25 flask with 2.5 mL of 0.1% gelatin and maintained in Gibco™ DMEM F12 medium (Thermo Fisher Scientific, Waltham, MA, USA, cat.12634010) supplemented with 40 µg mL^−1^ endothelial growth supplement (Corning, Corning, NY, USA, cat. # 356006). For the astrocyte and neuron cell lines, T-25 flasks were coated with 7.5 µL of poly-L-lysine in 7.5 mL of DI water (ScienCell™, Carlsbad, CA, USA, cat. # 1800-5). Astrocytes were maintained in Gibco™ complete medium (Thermo Fisher Scientific, Waltham, MA, USA, cat.A1261301), and neurons were maintained in complete Neuronal Medium (ScienCell™, Carlsbad, CA, USA, cat.# 1521).

### 2.2. EV Purification, Quantification, and Characterization

EVs were isolated from conditioned cell culture media using a commercial polymer precipitation kit (Thermo Fisher Scientific, Waltham, MA, USA, cat. 4478359). Three independent biological replicates per sample group were defined as separate cell culture preparations and EV isolations performed on different days. Conditioned cell media were collected from HEBCs, astrocytes, and neurons after 24 h of incubation and centrifuged at 3000× *g* for 10 min at 4 °C to remove cell debris. 5 mL of the polymer reagent was mixed with 10 mL of cleared media, vortexed, and incubated for 20 h at 4 °C. The media were centrifuged at 10,000× *g* for 1 h at 4 °C according to the vendor’s protocol. The supernatant was discarded, and the EV pellet was reconstituted using 500 µL of DPBS. Purified EVs were characterized by Nanoparticle Tracking Analysis (NTA), Human CD63 ELISA kit (Antibodies, St. Louis, MO, USA, cat. #A310656), and Human Calnexin kit (Antibodies, St. Louis, MO, USA, cat. #A312799). To assess co-precipitation of non-vesicular proteins with EVs, total protein in intact versus detergent-lysed EVs was measured using the Qubit Protein assay kit (Thermo Fisher Scientific, Waltham, MA, USA, cat. #Q33221). EVs were resuspended in 100 µL of PBS and lysed with M-PER™ Mammalian Protein Extraction Reagent (Thermo Fisher Scientific, Waltham, MA, USA, cat. #78501). A NanoSight Pro instrument (Malvern Pananalytical, Westborough, MA, USA) was used to perform NTA at an injection rate of 1.5 µL min^−1^ after diluting the EV samples 1:1 with DPBS. NS Xplorer software (Malvern Pananalytical, version 2.0) was used to analyze the concentration and size distribution of injected EV samples. The concentration of CD63 and calnexin was measured according to the ELISA vendor’s guidelines. 10 µg of protein equivalent from intact EVs was used per well for the assay, and protein concentration was determined by generating a standard curve. Three replicates were included for each experimental group.

### 2.3. timsTOF Pro 2 Mass Spectrometry Analysis

Proteins were extracted from EV samples using trifluoroacetic acid (TFA), followed by neutralization with Tris buffer [15]. Three biological replicates were included for each experimental group. 400 µL of TFA was added to 100 µL of the EV pellet, and the mixture was incubated for 60 s, then neutralized with 4 mL of 2 M Tris buffer. Subsequently, 440 µL of a 10× alkylation solution was added to each sample. The alkylation solution consisted of 100 mM Tris(2-carboxyethyl) phosphine (TCEP) and 400 mM 2-chloroacetamide (CAA) in deionized water. Total protein concentration after extraction was determined using the Pierce BCA Protein Assay Kit (Thermo Fisher Scientific, Waltham, MA, USA, Cat. #23225) according to the manufacturer’s instructions.

For proteolytic digestion, 7 µg of protein from each sample was digested with trypsin, and the resulting peptides were desalted using Pierce C18 Tips (Thermo Fisher Scientific, Waltham, MA, USA, Cat. #87782). A total of 50 ng of purified peptides was injected into a timsTOF Pro 2 mass spectrometer (Bruker Daltonics, Bremen, Germany) coupled to a Nano Elute LC system via a Captive Spray source. Peptides were separated on an in-house packed C18 column (75 µm × 15 cm, 1.9 µm; Dr. Maisch GmbH, Ammerbuch, Germany) at 40 °C, using 0.1% formic acid (FA) in water as mobile phase A and 0.1% FA in acetonitrile (ACN) as mobile phase B. Chromatographic separation was performed using a 21 min gradient: 2% to 30% B over 17.8 min, ramped to 95% B at 18.3 min, and held for an additional 2.4 min. The electrospray voltage was set to 1.5 kV, and the ion transfer tube temperature was maintained at 180 °C.

Mass spectrometric data were acquired in data-independent acquisition parallel accumulation–serial fragmentation (diaPASEF) mode using 16 *m*/*z* and ion mobility windows. Collision energy was ramped linearly as a function of ion mobility, from 20 eV at 1/K0 = 0.6 V·s·cm^−2^ to 59 eV at 1/K0 = 1.6 V·s·cm^−2^.

### 2.4. Data Processing and Analysis

Raw timsTOF Pro data were processed in Spectronaut (version 19.6, Biognosys, Newton, MA, USA) using the directDIA (DDI) workflow to generate a project-specific spectral library directly from the diaPASEF runs. Protein identification was performed against the UniProt human reference proteome at a 1% false discovery rate (FDR). Spectronaut performs protein-level statistical testing using Student’s *t*-tests across biological replicates and applies the Benjamini–Hochberg procedure to control the FDR. The statistically significant abundant proteins (*p* < 0.05 and log_2_ fold change ≥ 2.0) were categorized by GO biological process, molecular function, and cellular component using ShinyGO enrichment analysis (FDR < 0.00001) [16]. The proteins were organized into functional modules and included in Table 1.

## 3. Results

### 3.1. EV Characterization

**Figure 1:**
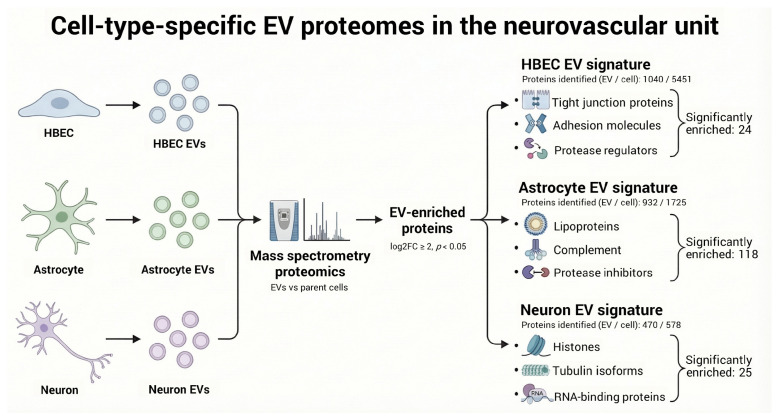
Experimental workflow for brain cell-derived EV proteome analysis. EVs were isolated from conditioned media of HEBCs, astrocytes, and neurons, and the vesicle and parent cell proteomes were analyzed by timsTOF Pro 2 mass spectrometry (log2 fold change ≥ 2, *p* < 0.05). The average number of proteins identified in EVs relative to parent cell lysates is shown for each cell type (HEBC: 1040/5451; Astrocyte: 932/1725; Neuron: 470/578). Significantly enriched EV proteins were categorized by functional module: HEBC EVs are enriched for tight junction proteins, adhesion molecules, and protease regulators (21 significantly enriched proteins); astrocyte EVs for lipoproteins, complement factors, and protease inhibitors (107 significantly enriched proteins); and neuronal EVs for histones, tubulin isoforms, and RNA-binding proteins (17 significantly enriched proteins).

Extracellular vesicles were isolated from the conditioned media of HEBCs, astrocytes, and neurons for comparative proteomic analysis of EVs and parent cell lysates. Figure 1 summarizes the experimental workflow, including cell culture, conditioned medium collection, timsTOF Pro mass spectrometry, the number of identified proteins, and an overview of major functional modules enriched in neuronal, astrocyte, and endothelial EV proteomes.

**Figure 2:**
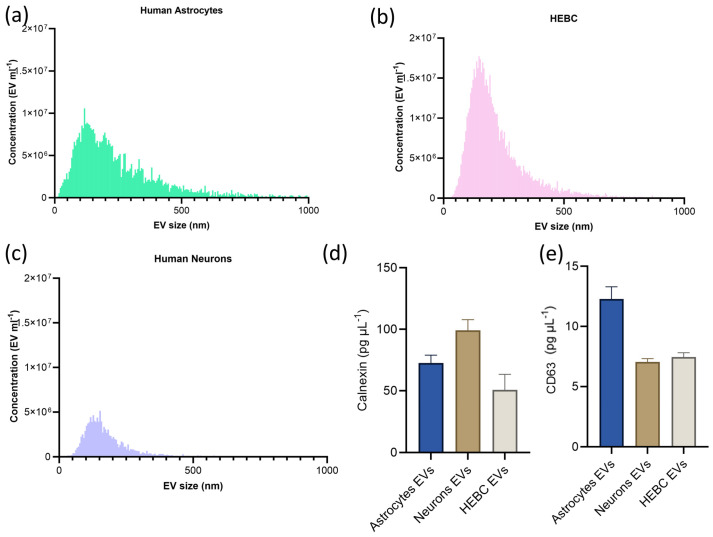
Size distribution and concentration of EVs purified from cell media of (**a**) human astrocytes, (**b**) HEBCs, and (**c**) human neurons. Each histogram is the average of three biological replicates. (**d**) Average concentration of CD63 and (**e**) calnexin in intact EVs. Each data point represents the mean of three biological replicates and the standard error of the mean.

Nanoparticle tracking analysis indicated a polydisperse size distribution across all three cell types, with the majority in the small EV range (80–150 nm) and a broader tail toward larger diameters, consistent with small, medium, and large EVs. Astrocyte-derived EVs were present at an average concentration of 4.82 × 10^8^ particles mL^−1^, with a modal diameter of 100 nm (Figure 2a), in agreement with the 107 nm peak size reported for human astrocyte EVs [17]. HEBC-derived EVs had a concentration of 1.05 × 10^9^ particles mL^−1^ and a modal diameter of 170 nm (Figure 2b), consistent with previously reported measurements [18]. Neuron-derived EVs had an average concentration of 2.08 × 10^8^ particles mL^−1^ and a modal size of 140 nm (Figure 2c), in agreement with NTA profiles for neuron-enriched EVs [19]. Astrocyte EVs contained on average 0.19 µg µL^−1^ protein when intact and 1026 µg µL^−1^ after lysis (*p* ≤ 0.05). Neuronal EVs showed a similar pattern, 0.40 µg µL^−1^ in intact and 1044 µg µL^−1^ after lysis. HEBC-derived EVs had 0.425 µg µL^−1^ and 1474 µg µL^−1^ before and after lysis, respectively. The significant increase in protein concentration after lysis indicates that the majority of detectable protein is membrane-protected in these fractions, consistent with protein packaging into vesicles; however, this assay does not exclude the presence of surface-associated proteins or co-precipitated non-vesicular material.

EV characterization by CD63 ELISA confirmed surface expression of the tetraspanin EV marker CD63 across all three cell types (Figure 2d). HEBC-derived EVs exhibited the highest CD63 concentration, consistent with their higher particle counts measured by NTA (1.05 × 10^9^ particles mL^−1^). In contrast, neuronal EVs showed the lowest CD63 levels, consistent with their comparatively low particle yield (2.08 × 10^8^ particles mL^−1^). Calnexin is associated with transmembrane, lipid, and intracellular compartments and is reported to be released with larger EVs in the 100–400 nm range [20]. Calnexin is associated with intracellular compartments and has been reported to be released in larger EVs in the 100–400 nm range [20]. Although calnexin was detected by ELISA in all three EV preparations (Figure 2e), it was not detected in the EV mass spectrometry datasets (File S2). This pattern indicates that intracellular- or large EV-associated calnexin is present at low abundance, sufficient to be detected by ELISA, but below the detection or reporting threshold of the mass spectrometer—consistent with a limited rather than dominant contribution of endoplasmic reticulum-derived contaminants.

The cell-type differences in EV yield likely reflect differences in secretory pathway activity and membrane turnover rates. Endothelial cells, which actively maintain the blood–brain barrier through continuous vesicular trafficking, would be expected to produce more EVs than post-mitotic neurons.

### 3.2. Proteome Coverage

**Figure 3:**
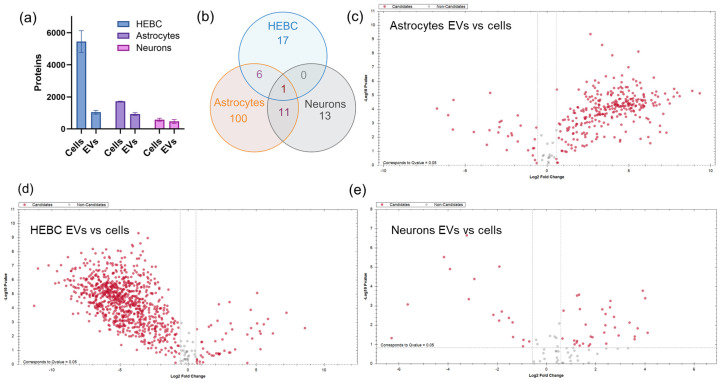
Proteome coverage and abundance across brain cell types. (**a**) Number of proteins identified in whole-cell lysates and in corresponding EVs from HEBCs, astrocytes, and neurons (mean ± SEM, n = 3). (**b**) Venn diagram showing overlap of EV proteins significantly abundant (log_2_ fold change ≥ 2.0, Benjamini–Hochberg-adjusted *p* < 0.05) among HEBC-, astrocyte-, and neuron-derived EVs. (**c**–**e**) Volcano plots comparing EVs to parent cells for astrocytes (**c**), HEBCs (**d**), and neurons (**e**).

Mass spectrometry analysis identified, on average, 932 proteins in astrocyte-derived EVs and 1725 in the corresponding total cell lysate. HEBC-derived EVs identified 1040 proteins, compared with 5451 in the corresponding cell lysate, while neurons had 578 proteins in cell lysates and 470 in EV cargo on average. Stringent enrichment criteria (log_2_ fold-change ≥ 2.0, adjusted *p* < 0.05) were applied to the raw mass spectrometry data, identifying 118 significantly enriched EV proteins in astrocytes, 24 in HEBCs, and 25 in neurons (Figure 3a). The high number of enriched astrocyte EV proteins (107, compared with 21 for HEBCs and 17 for neurons) suggests that astrocytes package a broad set of proteins, including lipoproteins, complement factors, and extracellular matrix components, into their EVs. The smaller enriched sets for HEBCs and neurons indicate more focused EV cargo specialization, such as protease regulation and hemostasis (HEBCs) or on chromatin-associated and cytoskeletal proteins (neurons).

Venn diagrams illustrate the extent of proteome overlap among the three cell types (Figure 3b). Complement component C3 was the only protein significantly enriched in EVs from all three cell types, suggesting a conserved role for EV-mediated complement signaling across the neurovascular unit. Its universal EV enrichment suggests that complement surveillance may be a fundamental feature of healthy intercellular communication in the brain, rather than an exclusive marker of pathology. Astrocyte- and neuron-derived EVs shared 11 enriched proteins, including nine histone H2B cluster members (H2BC4, H2BC5, H2BC9, H2BC12, H2BC13, H2BC14, H2BC15, H2BC18, and H2BS1), ALB, and the iron-binding protein LTF. The shared histone enrichment between astrocytes and neurons suggests that packaging of chromatin-associated proteins into EVs may indicate high transcriptional activity and chromatin remodeling. Astrocytes and HEBCs shared six proteins (APOA1, APOB, APOE, C7, SERPINC1, and THBS1), reflecting roles in lipid transport and coagulation/complement regulation at the blood–brain interface. The co-enrichment of three major apolipoproteins (APOA1, APOB, APOE) in both astrocyte and HEBC EVs is consistent with coordinated lipid shuttling across the glia–vascular interface, where astrocytes are the primary CNS producers of APOE-containing lipoproteins and endothelial cells regulate their transcytosis across the BBB. The absence of any shared enriched proteins exclusively between neurons and HEBCs suggests that neuron–endothelial EV communication operates through non-overlapping molecular pathways distinct from those governing the astrocyte–endothelial and astrocyte–neuronal axes.

### 3.3. Cell-Type-Specific EV-Enriched Proteins

**Figure 4:**
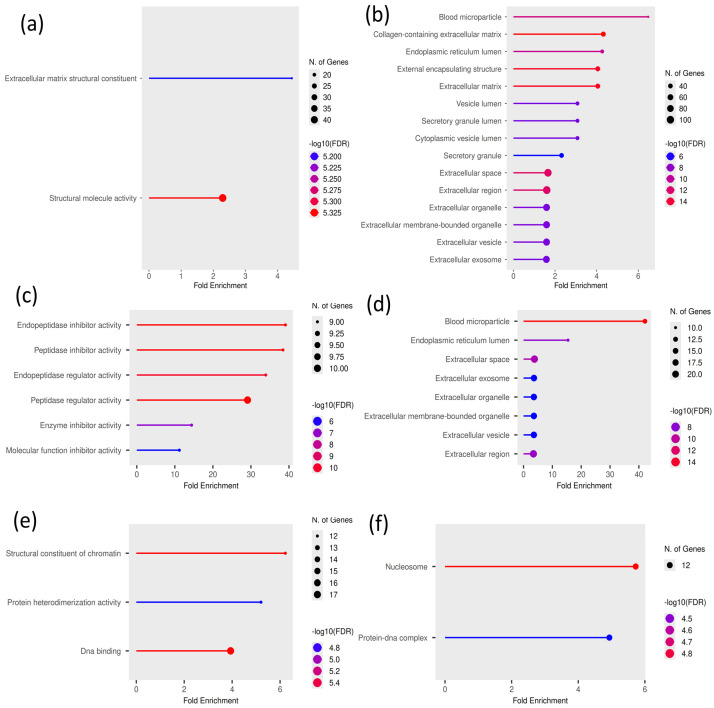
GO enrichment analysis was performed on proteins significantly enriched in EVs relative to parent-cell proteomes (log_2_ fold-change ≥ 2.0, *p* < 0.05). (**a**,**b**) Astrocyte-derived EVs show enrichment for extracellular matrix structural constituent and structural molecule activity terms, with cellular component categories including extracellular vesicles, extracellular space, and collagen-containing extracellular matrix. (**c**,**d**) HEBC-derived EVs are enriched for peptidase and endopeptidase inhibitor and regulator activities, as well as broader enzyme inhibitor functions, with cellular component terms including blood microparticle, extracellular vesicle/exosome, and extracellular matrix (**e**,**f**). Neuronal EVs are enriched for structural components of chromatin, protein heterodimerization activity, and DNA binding, with cellular component categories including nucleosomes, protein–DNA complexes, and extracellular vesicles. No extracellular matrix GO terms were enriched in the neuronal EV fraction, consistent with the predominant role of astrocytes and endothelial cells in ECM production within the NVU.

Proteins were considered significantly enriched in EVs based on a log_2_ fold-change ≥ 2.0 and statistical significance relative to parent-cell proteomes (*p* < 0.05). Gene Ontology analysis indicated that EVs from each cell type are enriched for functional categories consistent with their established biological roles. Astrocyte-derived EVs were overrepresented in extracellular matrix and structural molecule activity terms within the molecular function GO categories. In contrast, cellular component categories were enriched for extracellular vesicles, extracellular space, and collagen-containing extracellular matrix (Figure 4a,b). This enrichment pattern indicates that astrocyte EVs are actively loaded with extracellular matrix-remodeling proteins that support the glia–vascular interface. The presence of protease inhibitors (SERPINC1, SERPINE1, SERPINB3, SERPINF2, A2M) alongside basement membrane structural proteins (LAMB1, LAMB2, DAG1) suggests that astrocyte EVs simultaneously deliver matrix components and protective factors that prevent premature degradation of the perivascular extracellular matrix.

HEBC-derived EVs were enriched for molecular function terms, including peptidase and endopeptidase inhibitor and regulator activities, and for cellular component terms, including blood microparticle, extracellular vesicle, and extracellular matrix categories (Figure 4c,d). The enrichment of multiple SERPIN family members (SERPINA7, SERPINC1, SERPINF1) and coagulation components (F2, F10, PLG) in HEBC EVs suggests that endothelial cells could deploy EVs as a delivery system for anti-proteolytic defense. The co-packaging of hemostatic regulators (VWF, F2, F10) with complement components (C4A/C4B, C5, C7) suggests that HEBC EVs could coordinate crosstalk between coagulation and innate immunity, as simultaneous activation of both pathways drives thromboinflammation, a recognized mechanism of BBB disruption in neurodegeneration [21].

Neuronal EVs have a distinct profile compared with the other two cell types. GO molecular function categories were dominated by structural constituents of chromatin, protein heterodimerization activity, and DNA binding, and cellular component terms were enriched for nucleosome, protein–DNA complex, and extracellular vesicle terms (Figure 4e,f). The enrichment of nine histone H2B isoforms and protein heterodimerization activity is consistent with the packaging of intact or partially assembled nucleosomal complexes. The selective enrichment of H2B variants—but not core histones H3 or H4—suggests a specific packaging mechanism, possibly involving the endosomal sorting complexes required for transport pathway, which has been implicated in histone sorting into EVs [22]. The enrichment of the RNA-binding proteins HNRNPK and HNRNPA1 suggests that neuronal EVs may co-package chromatin-modifying factors with mRNA-processing proteins. Neuronal EVs did not enrich any extracellular matrix GO terms, in contrast to the ECM enrichment observed in astrocyte and HEBC EVs. This absence is consistent with the established cell biology of the CNS: astrocytes and other glial cells—not neurons—are the principal producers and secretors of ECM structural components. While neurons do contribute to specialized ECM structures such as perineuronal nets (PNNs), PNN assembly requires multicellular cooperation with glial-derived components and maturation signals that are absent in monoculture conditions [23]. The human neurons used in this study, cultured in the absence of astrocyte-derived signals, would not be expected to produce substantial ECM cargo for EV packaging. Proteomics studies of neuron-derived EVs have reported that their cargo is dominated by synaptic, cytoskeletal, and signaling proteins, with little enrichment for extracellular matrix [24].

### 3.4. Comparison with Curated EV Protein Databases

To benchmark the EV proteomes against established EV resources, we compared proteins identified in HEBC-, astrocyte-, and neuron-derived EVs with the Vesiclepedia Top 100 EV protein list. This analysis showed substantial overlap across all three EV preparations, supporting the EV identity of the isolated fractions. Astrocyte EVs contained 78 of the Top 100 EV proteins, HEBCs EVs overlapped with 81 of the Top 100 EV proteins, and neuron-derived EVs included 52 of the Top 100 EV proteins. These results indicate that EVs from all three neurovascular unit cell types share a large fraction of canonical EV markers while maintaining unique cell-type-specific cargo.

### 3.5. Comparative Proteomic Signatures Across Cell Types

A comparative analysis of statistically significant abundant EV proteins revealed cell-type-specific signatures. The proteins were grouped by functional module and compared across the three cell types (Table 1). Astrocyte EVs were enriched for extracellular matrix proteins, protease inhibitors, and lipoproteins. HEBC EVs were specialized for hemostasis and lipid metabolism, whereas neuronal EVs were enriched for chromatin-associated histones, cytoskeletal proteins, and RNA-binding factors (Table 1).

## 4. Discussion

This study establishes a baseline for EV-derived proteins from three major NVU cell types under healthy in vitro conditions. More significantly enriched EV proteins were identified in astrocytes (118) than in HEBCs (24) or neurons (25), reflecting differences in EV secretion rates and parent cell proteome complexity. Parallel analysis of EV and parent-cell proteomes identified proteins selectively packaged into EVs, which correlates with the functional roles of these cells. Astrocyte-derived EVs are enriched in apolipoproteins (APOB, APOA1, APOE), complement factors (C3, C7), and protease inhibitors (ITIH2/3/4, SERPINF2, SERPINC1), reflecting active metabolic and immunoregulatory roles. Several EV-enriched modules identified here are consistent with prior brain-derived EV proteomics. Human neural cell type-specific EV profiling and neural precursor cell-derived EV studies report astrocyte and neuron EV cargo enriched in apolipoproteins (including APOE and APOA1), complement components, cytoskeletal proteins, and RNA-binding factors, which overlap with the lipoprotein/complement and chromatin/cytoskeletal/RNA-binding modules listed in Table 1 [10,11]. Endothelial and vascular secretome (EV) proteome analyses identify extracellular matrix proteins, thrombospondin-1 (THBS1), coagulation factors, and SERPIN family members in secreted vesicles, consistent with the hemostatic and anti-proteolytic signature we observe in HEBC-derived EVs [25]. Several independent studies have demonstrated that APOE is a component of brain cell–derived EVs rather than a lipoprotein contaminant. APOE has been detected in astrocyte EVs, where it modulates microglial phenotypes and disease features in neuroinflammatory models [11,26]. The enrichment of APOE in the astrocyte- and HEBC-derived EV fractions likely reflects cell-type-specific EV loading of APOE-containing lipoprotein particles or APOE bound at the EV surface, rather than nonspecific carryover alone. APOE isoform-specific enrichment is significant because the APOE4 genotype is the strongest genetic risk factor for Alzheimer’s disease. Measuring APOE4 protein in brain-derived EVs could provide a dynamic, cell-type-specific readout of APOE4 abundance and secretion, improving risk stratification and enabling pharmacodynamic monitoring in trials targeting APOE or EV pathways. HEBC-derived EVs are associated with proteins involved in vascular homeostasis, including peptidase inhibitors (SERPINA7, SERPINC1, SERPINF1), coagulation factors (F2, F10, PLG), and extracellular matrix proteins (FN1, VTN, THBS1, COL isoforms). The co-enrichment of SERPIN family members with hemostatic regulators in HEBC EVs suggests a potential role in the coordinated suppression of pathological proteolysis, warranting direct functional validation in future studies. The enrichment of apolipoproteins in HEBC- and astrocyte-derived EVs suggests these cells are CNS lipid transport hubs that regulate neuronal lipid homeostasis and apolipoprotein metabolism. Neuronal EVs are enriched in histone H2B isoforms, neuronal markers, and RNA-binding proteins (HNRNPK, HNRNPA1, HNRNPU), suggesting dual roles in synaptic signaling and, potentially, in epigenetic intercellular communication [27]. The enrichment of tubulin isoforms (TUBB, TUBB3) and vimentin reflects active synaptic remodeling and cytoskeletal dynamics, processes linked to synaptic plasticity [28]. The EV proteomes from all three brain cell types showed significant overlap with the Vesiclepedia Top 100 canonical EV proteins, with 52–81% of the reference EV markers present in each preparation.

Neuronal EVs are enriched with proteins involved in synaptic function processes that are disrupted early in Alzheimer’s disease (AD). Synaptic loss is a major contributor to cognitive decline, making synaptic proteins in neuronal EVs particularly valuable as early biomarkers. Astrocyte EVs are enriched in proteins involved in lipid metabolism and BBB maintenance, consistent with astrocytic contributions to neuroinflammation and BBB breakdown observed in AD progression. Complement component C3 showed differential EV enrichment across all three cell types: 7-fold in astrocyte EVs, 5-fold in HEBCs, and 4-fold in neurons relative to their parent cell proteomes. The highest enrichment in astrocytes is consistent with the established role of reactive astrocytes as the primary CNS source of C3. It suggests that astrocyte EVs may serve as a major vector for complement-mediated paracrine signaling under both homeostatic and pathological conditions [29]. In the context of Alzheimer’s disease, C3 is a major driver of neuroinflammation and complement-mediated synaptic elimination, processes that represent early pathological events preceding widespread neuronal death [30]. Recent work demonstrates that complement activation is initiated early in AD and correlates with synaptic degeneration [31]. The increased abundance of iron transport proteins in all three cell lines reflects coordinated CNS iron homeostasis. Iron accumulation and ferroptosis are emerging hallmarks of AD neurodegeneration that contribute to oxidative stress and neuronal loss [32]. Neuronal EVs are enriched in cytoskeletal proteins (TUBB, TUBB3, VIM), metabolic enzymes (ALDH9A1, PGAM1, PYGL), and RNA-binding proteins, reflecting active neuronal homeostasis. Dysregulation of these EV components could compromise neuronal cytoskeletal integrity and RNA metabolism, contributing to α-synuclein pathology, mitochondrial dysfunction, and axonal degeneration observed in Parkinson’s Disease (PD) [33]. The cytoskeletal disruption observed in PD includes destabilization of microtubules and intermediate filaments, aligning with the enrichment of tubulin and vimentin in neuronal EVs. Pathological changes in EV cargo could complement existing α–synuclein biomarkers, and profiling of neuronal EVs may help stratify patients by progression risk and monitor responses to disease-modifying therapies in PD. The enrichment of PNP and HNRNPK in neuronal EVs suggests roles in neuronal nucleotide metabolism (PNP) and in the transfer of RNA-binding and gene-regulatory proteins (HNRNPK) to modulate cell signaling in recipient cells. PNP catalyzes the reversible phosphorolysis of purine nucleosides, and its enrichment in neuronal EVs suggests a role in extracellular purine salvage, potentially modulating adenosine signaling. This pathway regulates synaptic transmission, neuroinflammation, and neuroprotection [34]. HNRNPK is a multifunctional RNA-binding protein that regulates mRNA splicing, stability, and translation, and its EV-mediated transfer could influence gene expression in recipient cells [35]. The co-enrichment of three SERPIN family members (SERPINA7, SERPINC1, SERPINF1), hemostatic regulators (F2/prothrombin, F10/Factor X, PLG/plasminogen, VWF), and complement components (C4A/C4B, C5, C7) in HEBC-derived EVs suggests a coordinated anti-proteolytic and hemostatic effect. SERPINC1 (antithrombin III) is the primary physiological inhibitor of thrombin, and its enrichment in HEBC EVs suggests that endothelial cells mediate anticoagulant activity through EVs [36]. SERPINF1 (pigment epithelium-derived factor, PEDF) is a potent antiangiogenic and neuroprotective factor that may represent a mechanism of endothelium-derived neuroprotection [37]. The co-packaging of coagulation factors (F2, F10) with their respective inhibitors (SERPINC1) within the same EV population suggests that HEBC EVs function as self-regulating hemostatic units, capable of delivering both procoagulant and anticoagulant factors to maintain vascular homeostasis. This is directly relevant to BBB pathology in neurodegeneration: elevated matrix metalloproteinases (MMP2, MMP9) and reduced tight junction protein abundance characterize BBB dysfunction in multiple neurodegenerative diseases, and disrupted coagulation-complement crosstalk amplifies neuroinflammatory cascades [38].

This study identifies EV-abundant, cell-type-specific protein signatures with distinct functional roles in neuronal communication. Several limitations should be considered. Although our quantitative EV-versus-parent-cell proteomics identifies proteins significantly enriched in EV fractions but does not distinguish luminal cargo from surface-associated or co-precipitated proteins. Apolipoproteins (APOE, APOA1, APOB), complement and coagulation factors (C3, C7, F2, F10, PLG, VWF), and nuclear or RNA-binding proteins (H2B isoforms, HNRNPK) are considered EV-associated candidates rather than definitively intraluminal cargo. Future work, including Western blot validation, will be required to determine the packaging mechanisms of these proteins. The experiments were performed in cell culture, which lacks the complex tissue architecture and multicellular signaling present in the intact brain, limiting generalization to in vivo pathology and plasma biomarker applications. The work identifies cell-type-specific EV protein signatures but does not capture the full diversity of sequence variant proteoforms that may contribute to EV functional heterogeneity. Future studies employing proteoform-aware or top-down proteomics approaches will be essential to build on these findings by identifying additional EV protein diversity. Gene Ontology and pathway enrichment analyses assign biological functions to the identified proteins. Direct functional validation through in vivo models is necessary to confirm the roles of these EV proteins in blood–brain barrier homeostasis, immune regulation, and synaptic signaling. Each cell line was cultured in optimized cell media. Proteins introduced by media supplements may co-precipitate with EVs and contribute to some of the quantitative differences observed between astrocyte-, endothelial-, and neuron-derived EV-enriched fractions. Many of the proteins in the reported dataset are intracellular or structural markers, well-established astrocyte and endothelial extracellular matrix and hemostatic regulators, and are unlikely to originate from the media. The study cannot fully exclude medium-dependent effects, and future work using serum-free and matched medium-only controls will be essential to disentangle medium composition from cell-intrinsic contributions to EV cargo. The broad size distributions observed by NTA indicate that the samples represent heterogeneous EV-containing fractions, likely including small, medium, and large EVs. Despite these limitations, this work provides a source for identifying candidate EV biomarkers of NVU function and for future mechanistic studies.

## 5. Conclusions

This proteomics study establishes a baseline EV signature from the main cell types of the NVU and identifies cell-specific biological functions associated with EV cargo. These functions include synaptic signaling, complement and immune regulation, lipid homeostasis, iron metabolism, and blood–brain barrier integrity. The overlap between enriched EV cargo and pathways implicated in neurodegenerative diseases in the literature suggests potential translational relevance that will require direct testing in disease models. This work provides a foundational proteomic baseline for EVs derived from healthy brain cells, supporting subsequent validation and the development of blood-based EV biomarker studies for noninvasive monitoring of CNS pathology, including neuroinflammation, synaptic dysfunction, and blood–brain barrier compromise.

## Figures and Tables

**Table 1:** EV-enriched proteins by cell type and functional module. Proteins meeting enrichment criteria (log_2_ fold-change ≥ 2.0, adjusted *p*-value < 0.05) for EVs versus whole-cell lysates. Proteins are grouped into functional categories based on GO and pathway annotations. Data file included in Supplementary File S1.

Functional Module	Neurons	HEBC	Astrocytes
Chromatin and Epigenetics	H2BC12, H2BS1, H2BC5, H2BC4, H2BC18, H2BC9, H2BC15, H2BC14, H2BC13	None	H2BC12, H2BS1, H2BC5, H2BC4, H2BC18, H2BC9, H2BC15, H2BC14, H2BC13, HMGB1
Cytoskeletal and Microtubule	TUBB, TUBB3, TUBA1B, TUBA1C, VIM	None	None
RNA Binding and mRNA Processing	HNRNPK, HNRNPA1	None	None
Lipoproteins and Lipid Metabolism	None	APOA1, APOE, APOB, CP	APOB, APOA1, APOE, LCN1
Complement and Coagulation Cascade	C3	C3, C4A/C4B, C5, C7, PLG, THBS1, F10	C3, C7, F2, F5, ITIH2, ITIH3, ITIH4,
Protease Inhibitors and Proteolytic Regulators	None	SERPINA7, SERPINC1, SERPINF1	SERPINF2, ITIH2, ITIH3, ITIH4, SERPINC1, SERPINE1, SERPINB3, A2M, GSN, CTSA, CTSD
Extracellular Matrix	None	THBS1, FN1, FBLN1	COL1A1, COL4A2, COL6A3, LAMA4, LAMA5, LAMB1, LAMB2, TNC, VCAN, POSTN, SPARC, MFGE8, NCAN, GPC4, AGRN, THBS1, FN1
Metabolic Enzymes and Energy	PNP, EEF1G, PPIA, ALDH9A1, HSP90AA1	None	PGAM1, ATP2A2, PYGL, PLOD1, PLOD3, ASAH1, BLVRB, OLFM4, GGH, EPRS1
Growth Factors and Signaling/Innate Immunity	None	MAP3K2	TGFB2, CCN1, CCN2, IGF2R, IGFBP7, NCAN, AGRN, NTN1
Cell Adhesion and Junction	None	FBLN1, KIF13B,	BSG, DAG1, LAMB1, LAMB2, ITGA6, ITGB1
Iron transport	LTF, ALB	HBE1, HBG1, HBG2, CP	LTF, TF, HPX, HBA1, HBB, TTR

## Data Availability

The mass spectrometry proteomics data have been deposited to the ProteomeXchange Consortium (https://proteomecentral.proteomexchange.org, accessed on 17 January 2026) via the iProX partner repository with the dataset identifier PXD073186.

## Supplementary Materials

The following supporting information can be downloaded at: https://www.mdpi.com/article/10.3390/proteomes14020024/s1, File S1: Differentially abundant EVs; File S2: All EVs.
